# 

**Published:** 1887-08

**Authors:** 


CONTENTS:

ARTICLE I.—Epileptiform Neuralgia of the Fifth Nerve.
By Professor Victor Horsley ........................ 145

" II.—The Importance of a Liberal Professional Education in the Practice of Dental and Oral Surgery.
By Dr. G. Frank Lydston, M. D ........................ 159

" III.—The Preparation of Tissues for Examination with the Microscope with Special Reference to the Microscopical Examination of the Teeth.
By S. Flexner, Ph. G........................ 170

" IV.—Erythroxylon Coca and its Alkaloid—Cocaine.
By W. E. Baxter........................ 177

International Medical Congress........................ 182

Conditions of Membership in the Ninth International Medical Congress. 184

EDITORIAL, ETC.

Southern Dental Association........................ 185

OBITUARY.

Dr. J. R. Walker........................ 190

Ralph C. Purnell, M. D., D. D. S........................ 190

MONTHLY SUMMARY.

Temperature of the Human Skin........................ 191

